# Multinational trends in sepsis mortality between 1985 and 2019: a temporal analysis of the WHO Mortality Database

**DOI:** 10.1136/bmjopen-2023-074822

**Published:** 2024-09-12

**Authors:** Matthieu Komorowski, Justin D Salciccioli, Joseph Shalhoub, Anthony C Gordon, Dominic C Marshall

**Affiliations:** 1 Department of Surgery and Cancer, Imperial College London, London, UK; 2 Imperial College Healthcare NHS Trust, London, UK; 3 Pulmonary and Critical Care Medicine, Brigham and Women's Hospital, Boston, Massachusetts, USA; 4 Section of Vascular Surgery, Imperial College London, London, UK; 5 Cleveland Clinic London, London, UK

**Keywords:** epidemiology, infectious diseases, intensive & critical care

## Abstract

**Abstract:**

**Objectives:**

Understanding the burden of disease of sepsis is essential for monitoring the effectiveness of international strategies to improve sepsis care. Our objective was to describe the multinational trend of sepsis-related mortality for the period 1985–2019 from the WHO Mortality Database.

**Design:**

Retrospective analysis of the WHO Mortality Database.

**Setting:**

We included data from all countries defined by the WHO as having ‘high usability data’ and at least 10 years of total available data.

**Participants:**

From the WHO list of 50 countries with high usability data, 14 (28%) were excluded due to excessive missingness. We included and analysed data separately for male and female.

**Primary and secondary outcome measures:**

We analysed age-standardised mortality rates (ASMR) (weighted average of the age-specific mortality rates per 100 000 people, where the weights are the proportions of people in the corresponding age groups of the WHO standard population).

**Results:**

We included 1104 country-years worth of data from 36 countries with high usability data, accounting for around 15% of the world’s population. The median ASMR for men decreased from 37.8 deaths/100 000 (IQR 28.4–46.7) in 1985–1987 to 25.8 deaths/100 000 (IQR 19.2–37) in 2017–2019, an approximately 12% absolute (31.8% relative) decrease. For women, the overall ASMR decreased from 22.9 deaths/100 000 (IQR 17.7–32.2) to 16.2 deaths/100 000 (IQR 12.6–21.6), an approximately 6.7% absolute decrease (29.3% relative decrease). The analysis of country-level data revealed wide variations in estimates and trends.

**Conclusions:**

We observed a decrease in reported sepsis-related mortality across the majority of analysed nations between 1985 and 2019. However, significant variability remains between gender and health systems. System-level and population-level factors may contribute to these differences, and additional investigations are necessary to further explain these trends.

**STRENGTHS AND LIMITATIONS OF THIS STUDY**

- We describe sepsis-related age-standardised mortality rates (ASMR) for the period 1985–2019 and 36 countries, using the WHO Mortality Database, and include most of Europe, Israel, Australia, New Zealand, South Korea, Japan, Canada and the USA.
- We used joinpoint and locally weighted smoothing regression analysis to describe trends in ASMR.
- Mid-way through our analysis (in the year 2000), our data coverage represented about 15.7% of the world’s population (971 million out of 6.2 billion individuals).
- To limit the impact of changing sepsis definitions and International Classification of Diseases (ICD) taxonomies, we have not restricted ICD codes to ‘sepsis’ or ‘septicaemia’, but have also included the root causes of sepsis (such as pneumonia, peritonitis, etc) following previously described definitions.
- This approach does not capture well low-income and middle-income countries’ (LMICs) data.

## Background

 Sepsis affects between 25 and 50 million people annually worldwide, making it a leading cause of death and one of the most expensive conditions treated in hospitals.^1^,^4^ Challenges in sepsis management include early identification and diagnosis, severity classification and providing optimal targeted therapy.^2^ Since the early 1990s, various efforts have been made to improve early identification and standardise the definition of the sepsis syndrome.^5^,^7^

In 2017, the WHO declared sepsis ‘a global health priority’ and urged governments to ‘monitor incidence and outcomes from sepsis’.^8^ Understanding the burden of disease is essential for measuring the effectiveness of international strategies to improve sepsis care. Various publications have reported trends in sepsis incidence and mortality, although ongoing discrepancies in the definition of sepsis resulted in large variability in estimates the definition of sepsis and the methods for data extraction and preprocessing resulted in substantial variability in the reported incidence and outcomes.^3 9^ A uniform, consistent method has been called for, which would allow a more accurate evaluation of outcomes and clinical interventions across cohorts.^9^

Despite wide variation in national-level estimates of sepsis, most authors reported an increasing incidence over the last decades, along with decreasing trends in case mortality.^10^,^12^ Whether sepsis incidence has truly been increasing remains unclear, since different factors may have contributed to the trend, including increased awareness and varying definitions.^3 11^ In fact, an analysis by Rudd *et al*, using data from the Global Burden of Disease Study, suggested that the age-standardised sepsis incidence had fallen by 37% between 1990 and 2017.^3^ A meta-analysis of 170 sepsis studies (both interventional and observational) conducted between 2009 and 2019 only highlighted a decrease in 30-day septic shock mortality between 2009 and 2011, but not later.^13^

To provide comprehensive documentation of patterns in sepsis mortality trends, we used the WHO Mortality data from 36 countries with high usability data between 1985 and 2019 and reported country-level and multinational estimated sepsis-related deaths for both genders. We also analysed trends over time and performed sensitivity analyses using various sepsis definitions.

## Methods

### Study design and data source

We conducted an observational analysis of sepsis-related mortality using data deposited in the WHO Mortality Database (https://www.who.int/data/data-collection-tools/who-mortality-database; date of access: June 2022). This database records country-level data for deaths by age, sex and cause of death from 1955 onward, as reported annually by WHO member states through their local death registration systems. Of note, deaths are not attached to a particular clinical encounter or hospital admission, so mortality cannot be defined at a given time point (eg, ‘mortality at 90 days’). Primary causes of death are recorded using the International Classification of Diseases (ICD) classifications, which researchers have used in the past to abstract sepsis definitions. The WHO performs routine evaluation and verification of data deposited into the database and provides an assessment of overall data quality.^14^ We obtained sepsis-related, sex-specific mortality data from countries with ‘high usability data’, which reflects the overall quality of death registration data (see full list of countries in online supplemental material). We included countries with at least 10 years of total available data and excluded countries that did not satisfy these criteria.

### Data handling and statistical analyses

All analyses were done in SAS (V.9.4m5). Country-specific and sex-specific crude mortality data were extracted as raw SAS datafiles. For each country and year, we grouped data within the age groups provided by the World Standard Population, stratified by sex. Then, we calculated age-standardised mortality rates (ASMR) per 100 000 population using World Standard Population.^15^ We performed two different analyses.

First, for the assessment of overall trends in sepsis-related mortality, we performed locally weighted scatterplot smoother (LOESS) regression analysis of the average ASMR for all available countries. LOESS is a non-parametric technique which uses local weighted regression to fit a smooth curve through points in a scatter plot and can be useful to investigate trends for data without a known parametric form and robust fitting is necessary.^16^

Next, we performed a joinpoint regression analysis for each country individually, which estimates changes in linear slope for mortality trends over time.^17^ Briefly, it assesses the overall trends in mortality and identifies the best-fitting points where mortality rates change significantly (significant increase or significant decrease). The analysis initially starts with no joinpoints and tests for significant changes in the model with sequential addition of points where there is significant change in the slope of the line. Each joinpoint in the final model indicates a statistically significant change in mortality trend and Joinpoint software computes the annual percentage change for each piecewise trend by means of a generalised linear model, assuming Poisson distribution. Joinpoint regression was performed using SEER*Stat software V.8.3.6.0 provided by the US National Cancer Institute Surveillance Research Programme, and we have performed similar analyses previously using this data source and software.^18^,^20^ For the purpose of joinpoint analysis, we imputed missing data in a last observation carried forward manner.

### Sepsis definition

Sepsis is a generic clinical syndrome whose definitions have changed over time.^5^,^7^ Since the different sepsis definitions cannot be directly translated to ICD codes, various abstractions have been proposed by different authors including Martin, Angus and Flaatten, matching the current sepsis definition and versions 9 and 10 of the ICD taxonomy.^10 21 22^ While the ICD definitions required both infection criteria and organ dysfunction criteria, we only used the ‘infection criteria’ and assumed that any patient whose death certificate included a listed infection also had some organ dysfunction contributing to their death.

We chose the Angus definition for our main results over Martin and Flaatten definitions because it is more exhaustive and more widely used. Indeed, Angus’s abstraction relied on 105 or 150 infection codes, for the ICD-9 and ICD-10 versions, respectively (see online supplemental material). In comparison, Martin’s used only six or nine codes (for ICD-9 and ICD-10, respectively), whereas Flaatten used seven codes (only the ICD-10 version is available), so we did not implement those definitions (see online supplemental material).

### Patient and public involvement

Our research group includes a patient advisory group which directly informs our research priorities. The members of the panel confirmed being in agreement with the 2017 WHO Resolution WHA70.7 on ‘Improving the prevention, diagnosis and clinical management of sepsis’, which highlights the importance of public awareness of sepsis and estimating the global burden of sepsis.^8^

## Results

There were a total of 36 countries with sufficient data available during the period from 1985 to 2019 accounting for a total of 1104 country-years and 2208 potential data points from both genders for analysis, covering most of Europe, Israel, Australia, New Zealand, Japan, Canada and the USA. From the WHO list of 50 countries with high usability data, 14 (28%) were excluded due to excessive missingness. Near complete data were available for each country studied, with a total of 240 (9.8%) missing data elements. Mid-way through our analysis (in the year 2000), our data coverage represented about 15.7% of the world’s population (971 million out of 6.2 billion individuals).

### Multinational trends in sepsis mortality

**Figure 1:**
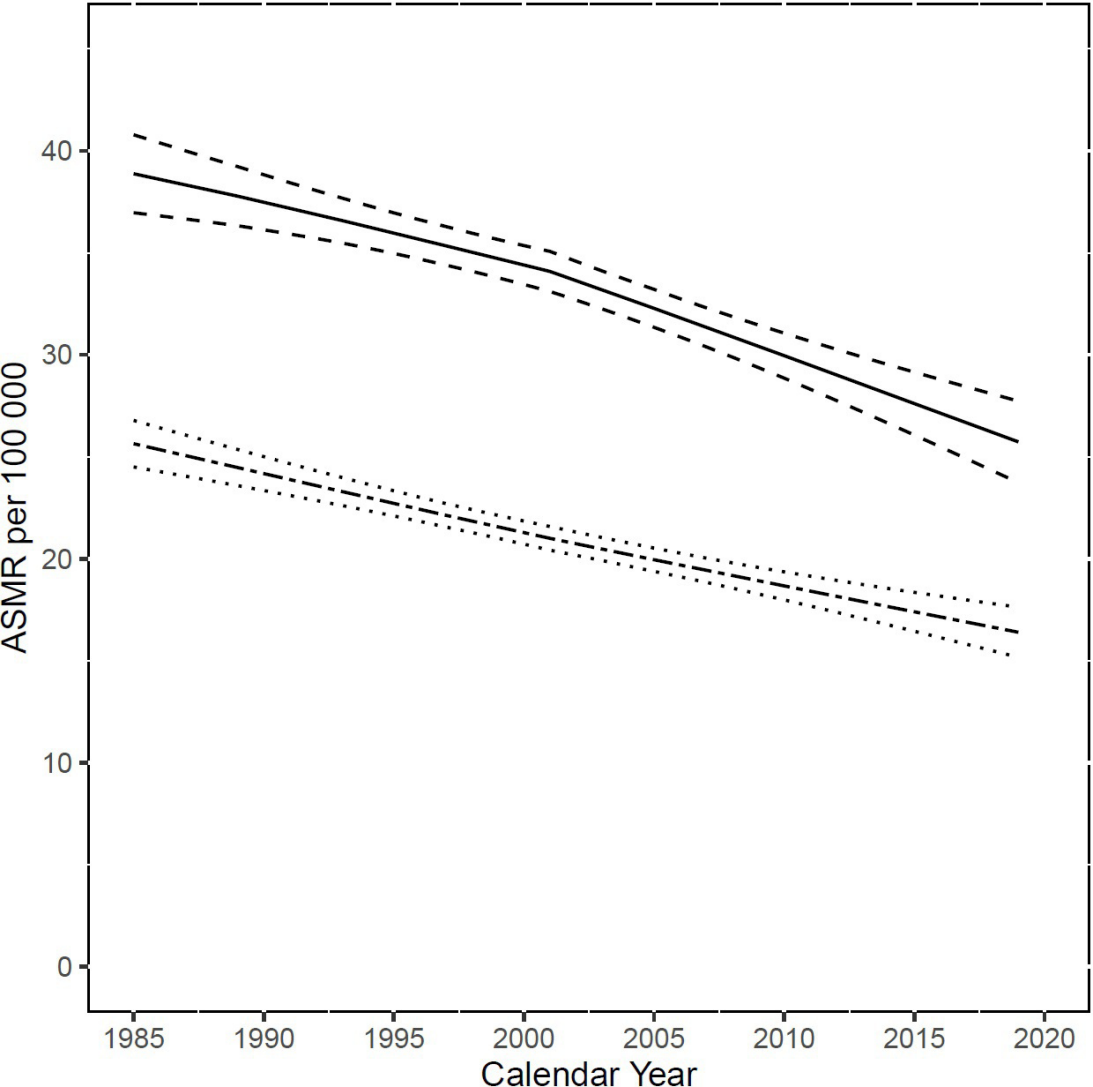
Multinational trend in age-standardised mortality rates (ASMR) of sepsis (Angus definition) per 100 000 population among the 36 included countries (weighted median±IQR), with LOESS regression. Full line represents male, dash-dotted line female. A decreasing trend in ASMR is observed for both genders, with ASMR decreasing faster in male.

Figure 1 presents the trend in sepsis mortality in all the included countries over the period 1985–2019, according to the Angus definition, with LOESS regression. In all countries, the ASMR was greater in men than women. The overall ASMR for men decreased from 37.8 deaths/100 000 (IQR 28.4–46.7) in 1985–1987 to 25.8 deaths/100 000 (IQR 19.2–37) in 2017–2019, an approximately 12% absolute (31.8% relative) decrease. For women, the overall ASMR decreased from 22.9 deaths/100 000 (IQR 17.7–32.2) to 16.2 deaths/100 000 (IQR 12.6–21.6), an approximately 6.7% absolute decrease (29.3% relative decrease) during the observation period. We measured a weak inverse correlation (R^2^ 0.25) between the ASMR during the early study period (1985–1987) and the observed relative change over the whole study period: countries with a higher early ASMR tend to be associated with a higher reduction in ASMR (see online supplemental material).

### Individual country trends in sepsis mortality

Table 1 shows ASMR per 100 000 population in 1985 and 2019 (or closest year available) for men and women, and the change between the start and the end. The top three countries with the lowest estimated ASMR in the end period were Austria, Finland and Slovenia for men, and Estonia, Finland and Slovenia for women. The highest mortality rates were demonstrated in Moldova, Poland and Romania, and Denmark, Romania and the UK for males and females, respectively. The majority of the 36 countries demonstrated a decrease in sepsis-related mortality over the study period. For men, the countries with the greatest percentage change over the observation period include Finland (−89%), Slovenia (−77%) and New Zealand (−71%), while the largest increases in mortality were observed in Malta (+31%), Denmark (+19%) and Greece (+16%). For women, the greatest percentage decreases were observed in Finland (−89%), Bulgaria (−75%) and Iceland (−74%), and the largest increases were observed in Greece (+45%), Malta (+34%) and Luxembourg (+27%).

**Table 1:** Average age-standardised mortality rates in 1985–1987 (start), in 2017–2019 (end) and absolute and relative change, for male and female

Country	Data period	Male			Female		
		Start	End	Change (%)	Start	End	Change (%)
Australia	1985–2018	19.72	17.24	−2.49 (−12.62)	16.1	14.46	−1.65 (−10.22)
Austria	1985–2019	30.33	13.8	−16.54 (−54.52)	20.21	9.95	−10.26 (−50.78)
Belgium	1985–2016	29.17	29.56	0.39 (1.34)	17.18	21.29	4.11 (23.91)
Bulgaria	1985–2018	71.37	25.01	−46.36 (−64.96)	49.32	12.22	−37.1 (−75.22)
Canada	1985–2005	30.84	21.53	−9.31 (−30.19)	19.26	17.96	−1.3 (−6.76)
Croatia	1985–2017	48.16	34.56	−13.6 (−28.24)	27.25	23.72	−3.53 (−12.95)
Czech Republic	1986–2019	46.53	37.15	−9.38 (−20.15)	36.16	24.42	−11.74 (−32.46)
Denmark	1994–2018	29.79	35.58	5.79 (19.43)	21.02	26.73	5.7 (27.13)
Estonia	1985–2016	36.16	23.42	−12.74 (−35.22)	15.83	9.74	−6.09 (−38.47)
Finland	1987–2018	51.07	5.25	−45.82 (−89.72)	31.77	3.54	−28.23 (−88.85)
France	1985–2014	26.5	18.33	−8.17 (−30.83)	15.68	11.31	−4.37 (−27.87)
Germany	1990–2019	28.22	24.2	−4.02 (−14.25)	18.72	16.66	−2.06 (−11.01)
Greece	1985–2018	17.4	20.27	2.87 (16.5)	13.48	19.63	6.15 (45.65)
Hungary	1985–2019	42.59	19.02	−23.57 (−55.35)	28.36	13.25	−15.11 (−53.27)
Iceland	1985–2019	53.36	16.56	−36.81 (−68.97)	54.17	13.85	−40.32 (−74.43)
Ireland	1985–2015	78.44	25.12	−53.32 (−67.98)	56.31	23.26	−33.05 (−58.69)
Israel	1985–2018	36.06	39.37	3.31 (9.19)	30.44	29.33	−1.1 (−3.63)
Italy	1985–2017	20.67	19.95	−0.72 (−3.47)	13.75	14.13	0.37 (2.71)
Japan	1985–2018	58.47	32.61	−25.85 (−44.22)	30.99	15.27	−15.72 (−50.73)
Latvia	1985–2018	35.96	33.59	−2.37 (−6.6)	19.41	14.62	−4.8 (−24.7)
Lithuania	1985–2019	35.34	39.55	4.22 (11.93)	16.65	18.05	1.4 (8.41)
Luxembourg	1985–2018	27.59	17.26	−10.32 (−37.42)	18.33	23.4	5.07 (27.64)
Malta	1985–2017	23.19	30.46	7.27 (31.34)	13.69	18.36	4.67 (34.11)
Moldova	1985–2018	69.48	47.16	−22.31 (−32.12)	39.16	16.07	−23.09 (−58.95)
Netherlands	1985–2018	26.19	22.16	−4.02 (−15.36)	20.63	18.15	−2.47 (−11.99)
New Zealand	1985–2016	55.01	15.83	−39.19 (−71.23)	41.9	16.58	−25.32 (−60.43)
Poland	1985–2018	41.39	44.34	2.94 (7.11)	23.95	22.39	−1.55 (−6.49)
Portugal	1985–2018	43.14	40.68	−2.46 (−5.71)	23.16	24.58	1.42 (6.15)
Romania	1985–2018	73.23	52.74	−20.49 (−27.98)	50.98	25.58	−25.4 (−49.82)
Slovakia	1992–2014	72.35	43.16	−29.19 (−40.35)	44.33	24.5	−19.83 (−44.73)
Slovenia	1985–2019	42.65	9.95	−32.7 (−76.68)	26.04	8.79	−17.24 (−66.23)
Spain	1985–2017	31.33	26.41	−4.92 (−15.71)	19.17	16.92	−2.25 (−11.72)
Sweden	1987–2018	40.23	18.61	−21.62 (−53.75)	27.58	13.44	−14.14 (−51.28)
Switzerland	1995–2019	19.12	15.43	−3.68 (−19.26)	13.44	11.41	−2.02 (−15.05)
United Kingdom	1985–2016	48.94	38.26	−10.68 (−21.82)	40.25	33.47	−6.78 (−16.84)
United States	1985–2007	37.95	28.35	−9.6 (−25.3)	24.29	23.73	−0.56 (−2.3)

We only report a percentage change for countries with complete start and end data.

NAnot available

**Figure 2:**
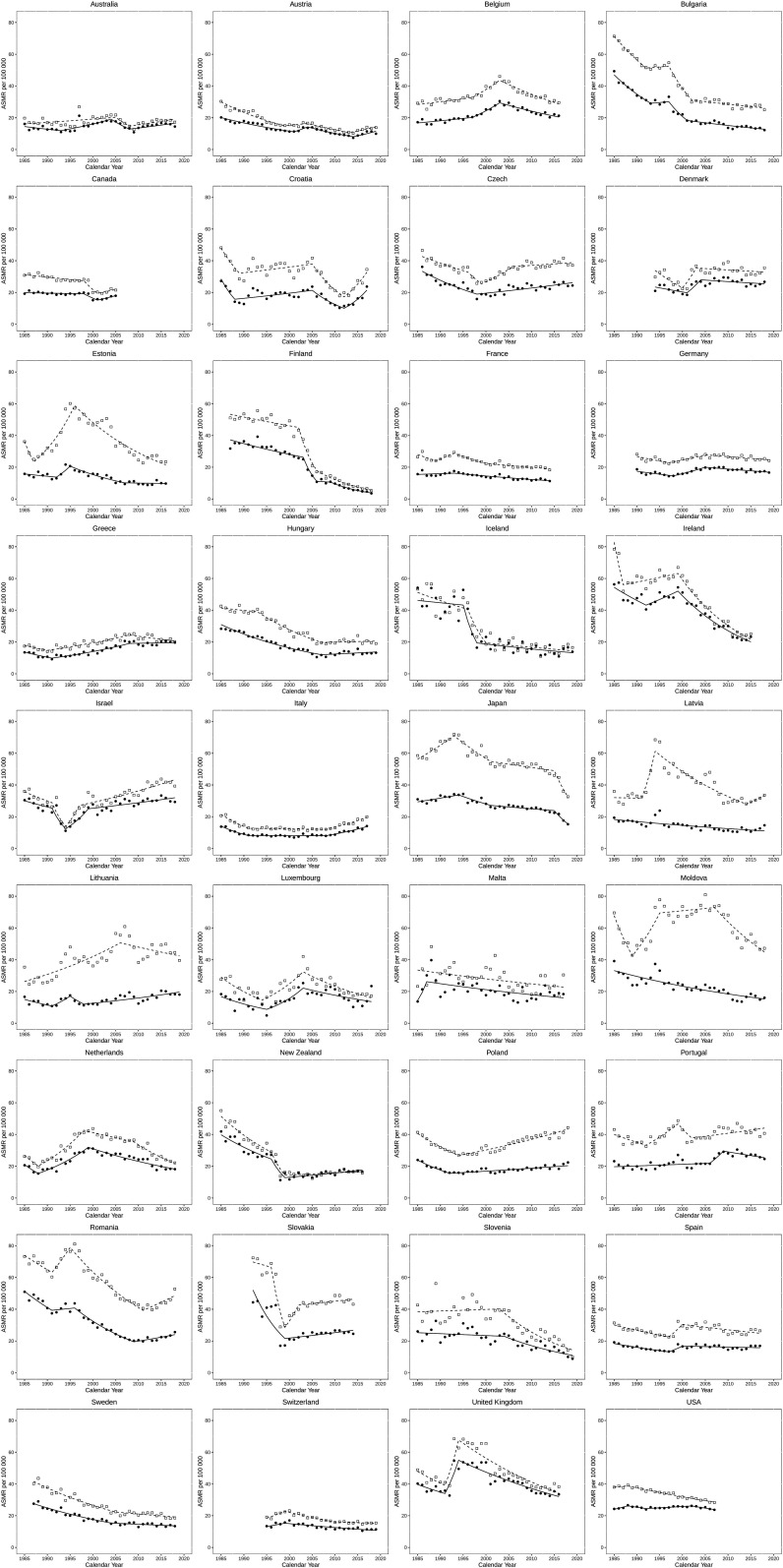
Age-standardised mortality rate (ASMR) of sepsis (Angus definition) and joinpoint regression analysis in 36 included countries between 1985 and 2019 (or longest period with data available). Empty squares represent male, full squares female.

Figure 2 shows trends in ASMR of sepsis according to the Angus definition, with joinpoint regression in all 36 included countries, for men and women. Many countries showed a decreasing trend (eg, Bulgaria, Hungary, Sweden, the USA), while some others had flat estimates (eg, Australia, Germany, Switzerland) or polyphasic trends (eg, Ireland, the Netherlands, the UK). In many eastern European countries (Estonia, Latvia, Lithuania, Moldova, Poland, Romania) as well as in Japan, the gender gap was striking with male mortality far exceeding that of female. Detailed numerical results of the joinpoint analysis for all individual countries is provided in online supplemental material.

## Discussion

In this work, we analysed the trends of sepsis-related mortality over 3 decades and 36 countries, representing one of the largest studies to date. Overall, we observed a decrease in reported sepsis-related mortality across the majority of analysed nations between 1985 and 2019, for both genders. However, there remains significant variability between health systems and genders with respect to trends in sepsis-related mortality.

A decrease in sepsis-related ASMR may reflect several factors, since ASMR combines both the incidence of a disease (which itself depends on disease definition and coding practices) as well as case fatality, which confounds the interpretation of the trends. Only the ASMR was available to us in the dataset we used, with no direct way of disentangling the relative contribution of incidence and case fatality. Many authors have reported an increase in the incidence and mortality rates of sepsis and septic shock in recent years, along with a significant decreasing trend in case fatality rates over time.^10^,^12^

In this situation, the ASMR in a given country can show different profiles. It can remain flat, if the increase in incidence compensates the decrease in case fatality. If the incidence increases more than the case fatality decreases, the ASMR will increase. Finally, the ASMR can decrease if the drop in case fatality exceeds the change in reported incidence. For example, a 50% decrease in case fatality in New Zealand between 2000 and 2012 was reported,^23^ while our analysis of the ASMR shows close to a flat line, which implies that the crude reported incidence may have doubled.

We confirmed that ASMR was consistently higher in males than females, which is mostly driven by a higher incidence of sepsis in men. Whether gender impacts sepsis survival is a matter of debate, with conflicting literature pointing either towards an increase or a reduction in mortality in women.^24 25^

Sepsis awareness has likely increased over the period of years, with efforts such as the Surviving Sepsis Campaign, Global Sepsis Alliance and World Sepsis Day.^26^,^28^ As a consequence, sepsis may be diagnosed and classified more frequently, which could have contributed to both the decrease in case fatality (by including more patients with lower levels of severity) and the increase in incidence. In 2020, Rudd *et al* reported the estimated global incidence and mortality of sepsis in 195 countries, using data from the Global Burden of Disease Study.^3^ Interestingly, they estimated a 37% decrease in age-standardised sepsis incidence over the 1990–2017 period, along with a 52.8% decrease in age-standardised mortality. Besides differences in data sources (Global Burden of Disease vs WHO Mortality Database), Rudd’s study used a regression modelling approach to make global estimates, while this study does not use modelling and assumes the ASMR obtained from the input data is globally representative.^29^ In this manuscript, the overall ASMR was significantly lower than in Rudd’s study, with rates of 21 per 100 000 in 2017/2019 compared with 148.1 per 100 000. The higher burden of sepsis in low-income and middle-income countries (LMICs), which were better represented in Rudd’s study, may have contributed to the differences between our estimates and theirs. Nevertheless, both studies revealed comparable findings, such as higher ASMR among males than females and a decline in ASMR over a similar time period. The estimates for specific countries were also strikingly similar in both studies, such as Australia, Israel, Croatia and Japan, which all differ by less than 5 per 100 000 across both studies. This highlights the value of the current study and the accuracy of using Angus’ definition in particular.

Our study has a number of limitations. The definition of sepsis and the version of the ICD taxonomy have changed over time. To limit the impact of this limitation, we have not restricted ICD codes to ‘sepsis’ or ‘septicaemia’ only but have also included the root causes of sepsis (such as pneumonia, peritonitis, etc) following previously described definitions based on ICD coding and presented sensitivity analyses from several definitions. Despite this, the transition point between versions of the ICD 9 and 10 nomenclatures are occasionally visible for some countries (eg, Iceland in 1996, Moldova in 1995 or New Zealand in 1999), which indicates that changes in ICD version have impacted our sepsis estimates.

We chose the Angus definition for our main results over Martin and Flaatten definitions because it is more exhaustive and more widely used. However, it has been reported that using the Angus definition may overestimate true sepsis incidence.^1^

In the Angus ICD abstraction of the sepsis definition, two criteria were required: a code for infection and a code for organ failure.^21 30^ Only the code for primary cause of death was available to us (as a suspected cause of death). We contend that since these patients died as a consequence of an infection, it is reasonable to assume that they indeed had sepsis and organ failure. The next limitation is that we relied on ICD coding, which has been frequently criticised for being unreliable.^1 31^ The practices of coding for death certificates vary across countries and within a county over time. For example, Rhee *et al* analysed sepsis incidence and mortality across 409 US hospitals, comparing clinical and claims data based on ICD-9-CM.^32^ They observed a large rise in incidence (a 50% increase between 2009 and 2014) based on ICD data, which was not reflected by clinical criteria (which remained essentially flat). The WHO itself acknowledges an uncertainty in the death rates ranging from ±10% for high-income countries to ±25%–35% for sub-Saharan Africa, pertaining to a combination of uncertainty in overall mortality levels, in cause of death assignment, and in the attribution of deaths coded to ill-defined causes.^33^ We only used countries with ‘high usability data’ as defined by the WHO itself.

We only presented aggregate national-level statistics. While our results provide valuable insights at a population level and allow for the examination of broad trends over time, they do not capture the full spectrum of variation that exists within populations, including differences in healthcare access, quality of care, socioeconomic factors and regional disparities. As such, our findings should be interpreted with caution, particularly when considering their direct applicability to individual clinical practice or policy-making.

Finally, this approach does not capture LMICs’ data well, since many of these countries have major gaps in data recording. This limits the coverage of the study, since 87% of the world’s population lives in LMICs. However, the validity of extrapolating data to these regions has been questioned.^3 8 29^

## Conclusions

Overall, we observed a decrease in reported sepsis-related mortality across the majority of analysed nations between 1985 and 2019, when relying on the definition by Angus *et al*. However, there remains significant variability between health systems with respect to trends in sepsis-related mortality. System-level and population-level factors may contribute to these differences in mortality and additional investigations are necessary to further explain these trends.

## supplementary material

10.1136/bmjopen-2023-074822 online supplemental file 1

## Data Availability

Data are available in a public, open access repository.
