# Supplementary material for: Multinational trends in sepsis mortality between 1985 and 2019: a temporal analysis of the WHO Mortality Database
Source: BMJ Open. 2024 Sep 12;14(9):e074822. doi: 10.1136/bmjopen-2023-074822 (PMC11404178; doi:10.1136/bmjopen-2023-074822)
Supplement: online supplemental file 1 [file bmjopen-14-9-s001.pdf]

Supplementary Material

To “Multinational Trends in Sepsis Mortality between 1985 and 2019: a temporal analysis of the WHO Mortality Database”

Authors: Matthieu Komorowski, Justin D Salciccioli, Joseph Shalhoub, Anthony C Gordon, Dominic C Marshall

Corresponding author: Matthieu Komorowski, Imperial College London, m.komorowski14@imperial.ac.uk

Table of Contents

Online Resource 1: List of high usability countries and included countries 2

Online Resource 2: Sepsis International Classification of Diseases (ICD) definitions 3

Martin, ICD-9 definition 6

Martin, ICD-10 definition 6

Flaatten, ICD-10 definition 7

Online Resource 3: Comparison of sepsis-related ASDR for 2001-2019, according to Angus, Martin and Flaatten definitions. 8

Online Resource 4: Relationship between the early ASDR for 1985-1987 and the change over the study period. 9

Online Resource 5: JoinPoint regression data for individual countries, Angus definition, period 1985-2019.. 10

Online Resource 1: List of high usability countries and included countries

Online Resource 1: List of countries with high usability data and list of countries included in the analysis

Area	Country	Included?
Americas	1. Canada<br>2. Dominica<br>3. Grenada<br>4. Mexico<br>5. Saint Vincent and the Grenadines<br>6. United States of America<br>7. Brazil<br>8. Chile<br>9. Cuba<br>10. Costa Rica	Yes<br>No<br>No<br>No<br>No<br>Yes<br>No<br>No<br>No<br>No
Europe	1. Andorra<br>2. Austria<br>3. Belgium<br>4. Croatia<br>5. Czechia<br>6. Denmark<br>7. Estonia<br>8. Finland<br>9. France<br>10. Germany<br>11. Hungary<br>12. Iceland<br>13. Ireland<br>14. Israel<br>15. Italy<br>16. Luxembourg<br>17. Malta<br>18. Netherlands<br>19. Norway<br>20. Romania<br>21. San Marino<br>22. Slovakia<br>23. Slovenia<br>24. Spain<br>25. Sweden<br>26. Switzerland<br>27. United Kingdom<br>28. Lithuania<br>29. The Former Yugoslav Republic of Macedonia<br>30. Kyrgyzstan<br>31. Latvia<br>32. Republic of Moldova<br>33. Uzbekistan	No<br>Yes<br>Yes<br>Yes<br>Yes<br>Yes<br>Yes<br>Yes<br>Yes<br>Yes<br>Yes<br>Yes<br>Yes<br>Yes<br>Yes<br>Yes<br>Yes<br>Yes<br>No<br>Yes<br>No<br>Yes<br>Yes<br>Yes<br>Yes<br>Yes<br>Yes<br>Yes<br>No<br><br>No<br>Yes<br>Yes<br>No
Western pacific region	1. Australia<br>2. Brunei<br>3. Darussalam<br>4. Japan<br>5. New Zealand<br>6. Republic of Korea	Yes<br>No<br>No<br>Yes<br>Yes<br>No
Africa	Mauritius	No

Online Resource 2: Sepsis International Classification of Diseases (ICD) definitions

As defined by	ICD-9	ICD-10
Angus	X	X
Martin	X	X
Flaatten	X	

Please note that we only used the "infection criteria", and not the "organ dysfunction" criteria. We assumed that any patient whose death certificate included a listed infection also had some organ dysfunction. Because Martin's and Flaatten's definitions relied on less than 10 "infection criteria" codes, we did not implement those sepsis abstractions in our research and used only Angus's. In Online Resource 4, we showed sepsis-related ASDR for 2001-2019 according to the three definitions and confirmed that Martin's and Flaatten's abstractions lead to unreliable results,

Angus, ICD-9 definition

Infection criteria (according to the ICD-9-Clinical Modification [CM] codes of Angus adjusted to ICD-9 Swedish version)—primary and secondary codes: 001, cholera; 002, typhoid/paratyphoid fever; 003, other salmonella infection; 004, shigellosis; 005, other food poisoning; 008, intestinal infection not otherwise classified; 009 ill-defined intestinal infection; 010, primary tuberculosis; 011, pulmonary tuberculosis; 012, other respiratory tuberculosis; 013, central nervous system tuberculosis; 014, intestinal tuberculosis; 015, tuberculosis of bone and joint; 016, genitourinary tuberculosis; 017, tuberculosis in other organs; 018, miliary tuberculosis; 020, plague; 021, tularemia; 022, anthrax; 023, brucellosis; 024, glanders; 025, melioidosis; 026, rat-bite fever; 027, other bacterial zoonoses; 030, leprosy; 031, other mycobacterial disease; 032, diphtheria; 033, whooping cough; 034, streptococcal throat/scarlet fever; 035, erysipelas; 036, meningococcal infection; 037, tetanus; 038, septicemia; 039, actinomycosis; 040, other bacterial diseases; 041, bacterial infection in other diseases not otherwise specified; 090, congenital syphilis; 091, early symptomatic syphilis; 092, early syphilis latent; 093, cardiovascular syphilis; 094, neurosyphilis; 095, other late symptomatic syphilis; 096, late syphilis latent; 097, other and unspecified syphilis; 098, gonococcal infections; 100, leptospirosis; 101, Vincent's angina; 102, yaws; 103, pinta; 104, other spirochetal infection; 110, dermatophytosis; 111, dermatomycosis not otherwise classified or specified; 112, candidiasis; 114, coccidioidomycosis; 115, histoplasmosis; 116, blastomycosis; 117, other mycosis; 118, opportunistic mycoses; 320, bacterial meningitis; 322, meningitis, unspecified; 324, central nervous system abscess; 325, phlebitis and thrombophlebitis of intracranial sinus; 420, acute pericarditis; 421, acute or subacute endocarditis; 451, phlebitis and thrombophlebitis; 461, acute sinusitis; 462 acute pharyngitis; 463, acute tonsillitis; 464, acute laryngitis/tracheitis; 465, acute upper respiratory infection of multiple sites/not otherwise specified; 481, pneumococcal pneumonia; 482, other bacterial pneumonia; 485, bronchopneumonia; 486, pneumonia, organism not otherwise specified; 491, chronic bronchitis; 494, bronchiectasis; 510, empyema; 513, lung/mediastinum abscess; 540, acute appendicitis; 541, appendicitis not otherwise specified; 542, other appendicitis; 562, diverticula of intestine; 566, anal and rectal abscess; 567,

peritonitis; 569F, intestinal abscess; 572A, abscess of liver; 572B, portal pyemia; 575A, acute cholecystitis; 590, kidney infection; 597, urethritis/urethral syndrome; 599A, urinary tract infection not otherwise specified; 601, prostatic inflammation; 614, female pelvic inflammation disease; 615, uterine inflammatory disease; 616, inflammatory disease of cervix, vagina, and vulva; 681, cellulitis, finger/toe; 682, other cellulitis and abscess; 683, acute lymphadenitis; 686, other local skin infection; 711A, pyogenic arthritis; 730, osteomyelitis and periostitis; 790H, bacteremia [WHO only has 790 Nonspecific findings on examination of blood]; 996G, infection or inflammation of device/graft; 998F, postoperative infection; 999D, infection, sepsis, or septicemia due to infusion, injection, transfusion, or vaccination.

Organ dysfunction criteria (according to the ICD-9-CM codes of Angus adjusted to ICD-9 Swedish version)— primary and secondary diagnoses: cardiovascular: 458, hypotension; 785F, shock without trauma. [WHO only has code 785: “Symptoms involving cardiovascular system”]
Respiratory: V46B, mechanical ventilation. Neurologic: 293, transient organic psychosis; 348B, anoxic brain damage; 348D, encephalopathy, unspecified. Hematologic: 286G, defibrination syndrome; 286x, other/unspecified coagulation defect; 287E, secondary thrombocytopenia [WHO only has code 287 Purpura and other haemorrhagic conditions]; 287F, thrombocytopenia, unspecified. Hepatic: 570, acute and subacute necrosis of liver; 573E, hepatic infarction. Kidney: 584, acute renal failure.

Angus, ICD-10 definition

Infection criteria (according to the ICD9-CM codes of Angus adjusted to ICD-10 Swedish version)— primary and secondary diagnoses: A00, cholera; A01, typhoid/paratyphoid fever; A02, other salmonella infection; A03, shigellosis; A04, other bacterial intestinal infections; A05, other bacterial foodborne intoxications, not elsewhere classified; A09, diarrhea and gastroenteritis of presumed infectious origin; A15, respiratory tuberculosis, bacteriologically and histologically confirmed; A16, respiratory tuberculosis, not confirmed bacteriologically or histologically; A17, tuberculosis of nervous system; A18, tuberculosis of other organs; A19, miliary tuberculosis, A20, plague; A21, tularemia; A22, anthrax; A23, brucellosis; A24, glanders and melioidosis; A25, rat-bite fevers; A26, erysipeloid; A27, leptospirosis; A28, other zoonotic bacterial diseases, not elsewhere classified; A30, leprosy; A31, infection due to other mycobacteria; A32, listeriosis; A34, obstetrical tetanus; A35, other tetanus; A36, diphtheria; A37, whooping cough; A38 scarlet fever; A39, meningococcal infection; A40, streptococcal septicemia; A41, other septicemia; A42, actinomycosis; A43, nocardiosis; A44, Bartonellosis; A46, erysipelas; A48, other bacterial diseases, not elsewhere classified; A49, bacterial infection of unspecified site; A50, congenital syphilis; A51, early syphilis; A52, late syphilis; A53, other and unspecified syphilis; A54, gonococcal infection; A55, chlamydial lymphogranuloma (venereum); A56, other sexually transmitted chlamydial diseases; A57, chancroid; A58, granuloma inguinale; A59, trichomoniasis; A65, nonvenereal syphilis; A66, yaws; A67, pinta; B35, dermatophytosis; B36, other superficial mycoses; B37, candidiasis; B38, coccidioidomycosis; B39, histoplasmosis; B40, blastomycosis; B41, paracoccidioidomycosis; B42, sporotrichosis; B43, chromomycosis and phaeomycotic abscess; B44, aspergillosis; B45, cryptococcosis; B46, zygomycosis; B47, mycetoma; B48, other mycoses, not elsewhere classified; B49, unspecified mycosis; B95, streptococcus and staphylococcus as the cause of diseases classified to other chapters; B96, other bacterial agents as the cause of diseases classified to other chapters; B97, viral agents as the

cause of diseases classified to other chapters; G00, bacterial meningitis, not elsewhere classified; G01, meningitis in bacterial diseases classified elsewhere; G02, meningitis in other infectious and parasitic diseases classified elsewhere; G03, meningitis due to other and unspecified causes; G04, encephalitis, myelitis and encephalomyelitis; G05, encephalitis, myelitis, and encephalomyelitis in diseases classified elsewhere; G07, intracranial and intraspinal abscess and granuloma in diseases classified elsewhere; G08, intracranial and intraspinal phlebitis and thrombophlebitis; I30, acute pericarditis; I33, acute pericarditis; I39.8, endocarditis, valve unspecified, in diseases classified elsewhere; I80, phlebitis and thrombophlebitis; J01, acute sinusitis; J02, acute pharyngitis; J03, acute tonsillitis; J04, acute laryngitis and tracheitis; J05, acute obstructive laryngitis (croup) and epiglottitis; J06, acute upper respiratory infections of multiple and unspecified sites; J13, pneumonia due to Streptococcus pneumoniae; J14, pneumonia due to Haemophilus influenzae; J15, bacterial pneumonia, not elsewhere classified; J18, pneumonia, organism unspecified; J20, acute bronchitis; J44.0, chronic obstructive pulmonary disease with acute lower respiratory infection; J44.1, chronic obstructive pulmonary disease with acute exacerbation, unspecified; J47, bronchiectasis; J80, adult respiratory distress syndrome; J85, abscess of lung and mediastinum; J86, pyothorax; J95.1, acute pulmonary insufficiency following thoracic surgery; J95.2, acute pulmonary insufficiency following nonthoracic surgery; J96.0, acute respiratory failure; J96.9, respiratory failure, unspecified; K35, acute appendicitis; K36, other appendicitis; K37, unspecified appendicitis; K57, diverticular disease of intestine; K61, abscess of anal and rectal regions; K63.0, abscess of the intestine; K63.1, perforation of intestine (nontraumatic); K65, peritonitis; K75.0, abscess of the liver; K75.1, phlebitis of portal vein; K81, cholecystitis; L02, cutaneous abscess, furuncle, and carbuncle; L03, cellulitis; L04, acute lymphadenitis; L08, other local infections of skin and subcutaneous tissue; M00, pyogenic arthritis; M86, osteomyelitis; N10, acute tubulointerstitial nephritis; N11, chronic tubulointerstitial nephritis; N12, tubulointerstitial nephritis, not specified as acute or chronic; N20.9, urinary calculus, unspecified; N30.0, acute cystitis; N30.1, interstitial cystitis (chronic); N30.2, other chronic cystitis; N30.3, trigonitis; N30.8, other cystitis; N30.9, cystitis, unspecified; N34.1, nonspecific urethritis; N41, inflammatory diseases of prostate; N70, salpingitis and oophoritis; N71, inflammatory disease of uterus, except cervix; N72, inflammatory disease of cervix uteri; N73, other female pelvic inflammatory diseases; N75, diseases of Bartholin's gland; N76, other inflammation of vagina and vulva; O23.0, infections of kidney in pregnancy; T80.2, infections following infusion, transfusion, and therapeutic injection; T81.4, infection following a procedure, not elsewhere classified; T81.6, acute reaction to foreign substance accidentally left during a procedure; T82.6, infection and inflammatory reaction due to cardiac valve prosthesis; T82.7, infection and inflammatory reaction due to other cardiac and vascular devices, implants, and grafts; T83.5, infection and inflammatory reaction due to prosthetic device, implant, and graft in urinary system; T83.6, infection and inflammatory reaction due to prosthetic device, implant, and graft in tge genital tract; T84.5, infection and inflammatory reaction due to internal joint prosthesis; T84.6, infection and inflammatory reaction due to internal fixation device; T84.7, infection and inflammatory reaction due to other internal orthopaedic prosthetic devices, implants, and grafts; T85.7, infection and inflammatory reaction due to other internal prosthetic devices, implants, and grafts; T88.0, infection following immunization.

Organ dysfunction criteria (according to the ICD-9-CM codes of Angus adjusted to ICD-10 Swedish version)—primary and secondary diagnoses: Cardiovascular: I95, hypotension; R57, shock, not elsewhere classified. Respiratory: Z99.1, dependence on respirator. Hematologic: D65, disseminated intravascular coagulation; D68.9, coagulation defect, unspecified; D69.5, secondary

thrombocytopenia; D69.6, thrombocytopenia, unspecified. Neurologic: F05.0, delirium not superimposed on dementia; F05.8, other delirium; F05.9, delirium, unspecified; G63.1, anoxic brain damage, not elsewhere classified; G93.4, encephalopathy, unspecified; R41, other symptoms and signs involving cognitive functions and awareness. Hepatic: K72.0, acute and subacute hepatic failure; K72.9, hepatic failure, unspecified; K76.2, central hemorrhagic necrosis of liver; K76.3, infarction of the liver. Kidney: N17, acute renal failure; N99.0, postprocedural renal failure.

Martin, ICD-9 definition

Infection criteria (according to the ICD-9-CM codes of Martin et al [18] adjusted to ICD-9 Swedish version)— primary and secondary diagnoses: 038, septicemia; 020, Plague; 790H, bacteremia; 117x, other and unspecified mycoses; 112F, disseminated candidiasis; 112W, candidiasis of other specified sites.

Organ dysfunction criteria (according to the ICD-9-CM codes of Martin et al [17] adjusted to ICD-9 Swedish version)—primary and secondary diagnoses: cardiovascular: 458A, orthostatic hypotension; 458x, hypotension, unspecified; 785F, shock without mention of trauma; 796D, nonspecific low blood pressure reading. Respiratory: 518F, pulmonary insuffi- ciency following trauma and surgery; 786A, dyspnea and respiratory abnormalities; 799B, respiratory arrest; V46B, mechanical ventilation. Hematologic: 286G, defibrination syndrome; 286x, other and unspecified coagulation defects; 287D, primary thrombocytepenia; 287E, secondary thrombocytopenia; 287F, thrombocytopenia, unspecified. Neurologic: 293, transient mental disorders due to conditions classified elsewhere; 348B, anoxic brain damage; 348D, encephalopathy, not elsewhere classified; 780A, alteration of consciousness. Kidney: 584, acute renal failure; 580, acute glomerulonephritis; 586, renal failure, unspecified; V56A, extracorporeal dialysis. Hepatic: 570, acute and subacute necrosis of the liver; 572C, hepatic coma. Metabolic: 276c, acidosis.

Martin, ICD-10 definition

Infection criteria (according to the ICD-9-CM codes of Martin adjusted to ICD-10 Swedish version)— primary and secondary diagnoses: A20, plague; A40, streptococcal septicemia; A41, other septicemia; A49.9, bacterial infection, unspecified; B49, unspecified mycosis; B37.5, candidal meningitis; B37.6, candidal endocarditis; B37.7, candidal septicemia; B37.8, candidiasis of other sites.

Organ dysfunction criteria (according to the ICD-9-CM codes of Martin adjusted to ICD-10 Swedish version)—primary and secondary diagnoses: cardiovascular: I95.1, orthostatic hypotension; I95.9, hypotension, unspecified; R03.1, nonspecific low blood-pressure reading; R57, shock, not elsewhere classified. Respiratory: J95.1, acute pulmonary insufficiency following thoracic surgery; J95.2, acute pulmonary insufficiency following nonthoracic surgery; J80, adult respiratory distress syndrome; R06.0, dyspnea; R06.3, periodic breathing; R06.4, hyperventilation; R06.8, other and unspecified abnormalities of breathing; R09.2, respiratory arrest; J96.0, acute respiratory failure; J96.9, respiratory failure, unspecified. Hematologic: D65, disseminated intravascular coagulation;

D68.9, coagulation defect, unspecified; D69.3, idiopathic thrombocytopenic purpura; D69.4, other primary thrombocytopenia; D69.5, secondary thrombocytopenia; D69.6, thrombocytopenia, unspecified. Neurologic: F05.0, delirium not superimposed on dementia; F05.8, other delirium; F05.9, delirium, unspecified; G93.1, anoxic brain damage, not elsewhere classified; G93.4, encephalopathy, unspecified; R40, somnolence, stupor and coma; R41.8, other and unspecified symptoms and signs involving cognitive functions and awareness; R41.0, disorientation, unspecified. Kidney: N00, acute nephritic syndrome; N01, rapidly progressive nephritic syndrome; N17, acute renal failure; N19, unspecified renal failure; Z49.1, extracorporeal dialysis. Hepatic: K72.0, acute and subacute hepatic failure; K72.9, hepatic failure, unspecified; K76.2, central hemorrhagic necrosis of liver. Metabolic: E87.2, acidosis.

Flaatten, ICD-10 definition

Infection criteria (according to the ICD-10 codes of Flaatten)—primary and secondary diagnoses: A26.7, Erysipelothrix septicemia; A39, meningococcal infection; A40.0, septicemia due to streptococcus, group A; A41, other septicemia; A42.7, actinomycotic septicemia; B37.7, candidal septicemia; T81.4, infection following a procedure, not elsewhere classified.

Organ dysfunction criteria (according to the ICD-10 codes of Flaatten)— only secondary diagnoses: cardiovascular: A41.9, septicemia, unspecified; I50.9, heart failure, unspecified. Respiratory: J13, pneumonia due to Streptococcus pneumoniae; J14, pneumonia due to Haemophilus influenzae; J15, bacterial pneumonia, not elsewhere classified; J16, pneumonia due to other infectious organisms, not elsewhere classified; J17, pneumonia in diseases classified elsewhere; J18, pneumonia, organism unspecified; J80, adult respiratory distress syndrome; J95, postprocedural respiratory disorders, not elsewhere classified; J96.0, acute respiratory failure. Renal: N17, acute renal failure; N99.0, postprocedural renal failure. Hematologic: D65, disseminated intravascular coagulation; D69, purpura and other hemorrhagic conditions. Other: E86, volume depletion; E87.2, acidosis; K72, hepatic failure, not elsewhere classified.

Online Resource 3: Comparison of sepsis-related ASDR for 2001-2019, according to Angus, Martin and Flaatten definitions.

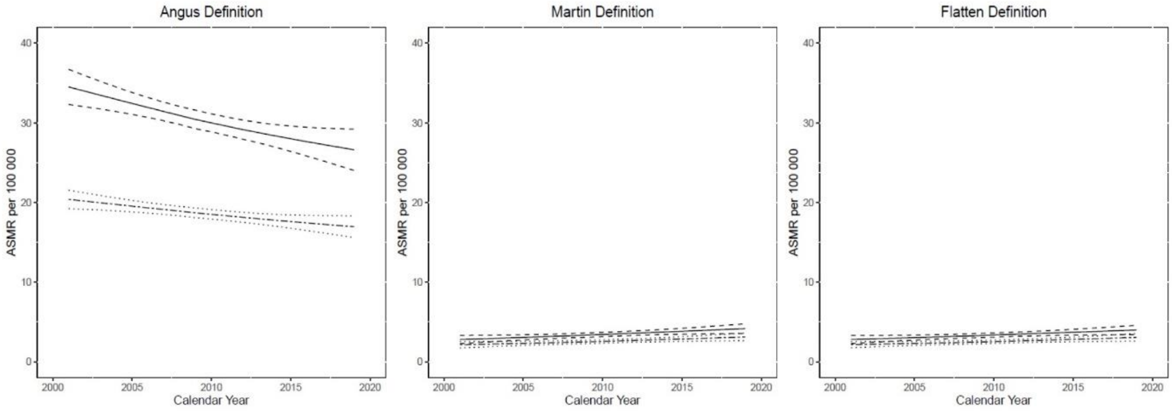

Online Resource 3: Comparison of three different sepsis abstractions. The plots represent sepsis-related ASDR among the 36 included countries (weighted median +/- IQR) according to Angus, Martin and Flaatten definitions, with LOESS regression, for 2001-2019. Dashdotted lines represent females, full lines males. Definitions lead to wide differences in sepsis estimates. We confirmed that Martin and Flaatten's definitions lead to unreliable estimates, because they rely on less than 10 ICD codes for the infection criteria.

Online Resource 4: Relationship between the early ASDR for 1985-1987 and the change over the study period.

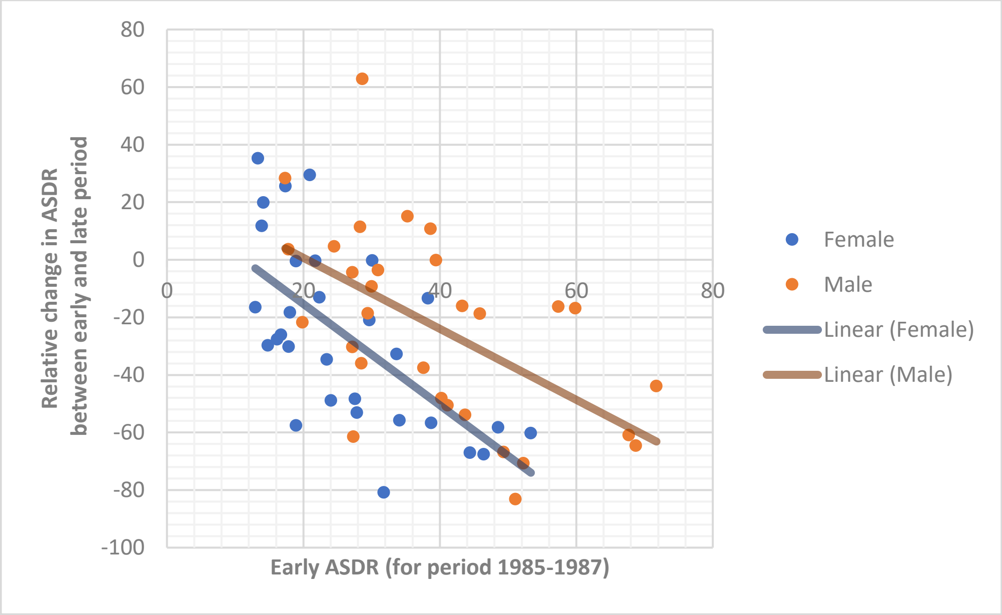

Online Resource 4: Relationship between the early ASDR (in the period 1985-1987) and the relative change over the study period, separating male in orange and female in dark blue. The linear regression trend lines are also shown. We excluded 6 countries which did not have data available for the start and/or end period. Overall, there is a weak (R coefficient -0.50 ; R-squared 0.25) inverse correlation between the ASDR during the early study period (1985-1987) and the observed relative change over the whole study period: countries with a higher early ASDR tend to be associated with a higher reduction in ASDR over the study period.

Online Resource 5: JoinPoint regression data for individual countries, Angus definition, period 1985-2019

Online Resource 5: Joinpoint regression analysis using the Angus definition, for male (table S2a) and female (table S2b) for all included countries over 1985-2019, or as indicated. We identified between one and four trends in each country.

Online Table 5a (Male):

Country	Trend 1		Trend 2		Trend 3		Trend 4	
	Years	APC	Years	APC	Years	APC	Years	APC
Australia	1985 - 2005	1.17^	2006 - 2008	-10.12	2009 - 2019	2.65		
Austria	1985 - 1999	-4.88^	2000 - 2004	1.97	2005 - 2013	-4.70^	2014 - 2020	4.99^
Belgium	1985 - 1997	1.21^	1998 - 2003	4.88^	2004 - 2017	-3.16^		
Bulgaria	1985 - 1992	-4.59^	1993 - 1997	0.46	1998 - 2002	-9.90^	2003 - 2019	-1.09^
Ca da	1985 - 1998	-1.01^	1999 - 2001	-10.74^	2002 - 2006	3.01		
Croatia	1985 - 1989	-9.47^	1990 - 2005	1.04	2006 - 2012	-10.63^	2013 - 2018	13.64^
Czech Republic	1986 - 1996	-2.57^	1997 - 1999	-8.15	2000 - 2006	5.19^	2007 - 2020	0.51
De ngk	1994 - 2000	-6.28^	2001 - 2003	15.55	2004 - 2019	-0.49		
Estonia	1985 - 1987	-19.71	1988 - 1996	10.86^	1997 - 2017	-4.90^		
Finland	1987 - 2002	-1.12^	2003 - 2006	-20.59^	2007 - 2019	-9.22^		
France	1985 - 1989	-3.73	1990 - 1993	4.6	1994 - 2000	-3.52^	2001 - 2015	-1.16^
Germany	1990 - 1997	-2.25^	1998 - 2005	2.47^	2006 - 2020	-0.82^		

Greece	1985 - 1990	-3.72	1991 - 2008	2.86^	2009 - 2019	-1.69^		
Hungary	1985 - 1993	-0.74	1994 - 2007	-4.75^	2008 - 2020	0.35		
Iceland	1985 - 1996	-2.1	1997 - 2000	-16.78	2001 - 2020	-1.54		
Ireland	1985 - 1987	-17.75	1988 - 1999	1.01	2000 - 2016	-6.47^		
Israel	1985 - 1991	-3.46	1992 - 1994	-21.60^	1995 - 1997	24.9	1998 - 2019	2.26^
Italy	1985 - 1991	-8.31^	1992 - 2008	-0.33	2009 - 2018	5.61^		
Japan	1985 - 1993	2.95^	1994 - 2001	-3.17^	2002 - 2015	-0.80^	2016 - 2019	-12.89^
Latvia	1985 - 1991	-0.21	1992 - 1994	24.61	1995 - 2014	-3.84^	2015 - 2019	4.5
Lithuania	1985 - 2006	3.16^	2007 - 2020	-1.35				
Luxembourg	1985 - 1994	-7.36^	1995 - 2003	9.27^	2004 - 2019	-4.60^		
Malta	1985 - 2018	-1.20^						
Moldova	1985 - 1989	-11.00^	1990 - 1995	8.53^	1996 - 2007	0.4	2008 - 2019	-4.31^
Netherlands	1985 - 1988	-9.02	1989 - 1998	7.77^	1999 - 2009	-1.69^	2010 - 2019	-5.28^
New Zealand	1985 - 1996	-5.12^	1997 - 1999	-20.74^	2000 - 2017	0.89^		
Poland	1985 - 1994	-4.53^	1995 - 2019	2.04^				
Portugal	1985 - 1992	-2.62^	1993 - 1999	4.98^	2000 - 2002	-6.8	2003 - 2019	0.96^
Romania	1985 - 1991	-2.58^	1992 - 1995	5.72	1996 - 2011	-4.27^	2012 - 2019	3.08^
Slovakia	1992 - 1996	-1.22	1997 - 1999	-24.54^	2000 - 2002	14.31	2003 - 2015	0.69
Slovenia	1985 - 2003	0.25	2004 - 2020	-6.24^				

Spain	1985 - 1997	-2.38^	1998 - 2000	10.58	2001 - 2018	-1.19^		
Sweden	1987 - 2005	-3.40^	2006 - 2019	-1.14^				
Switzerland	1995 - 1999	5.44^	2000 - 2010	-3.16^	2011 - 2020	-0.54		
United Kingdom	1985 - 1991	-3.29	1992 - 1994	20.02	1995 - 2017	-3.05^		
United States	1985 - 1988	0.65	1989 - 2008	-1.60^				

Online Table 5b (Female):

Country	Trend 1		Trend 2		Trend 3		Trend 4	
	Years	APC	Years	APC	Years	APC	Years	APC
Australia	1985 - 1993	-2.4	1994 - 2004	4.40^	2005 - 2008	-9.44	2009 - 2019	2.61
Austria	1985 - 2001	-3.64^	2002 - 2004	8.63	2005 - 2014	-5.53^	2015 - 2020	7.38^
Belgium	1985 - 1997	1.60^	1998 - 2003	6.45^	2004 - 2017	-2.76^		
Bulgaria	1985 - 1993	-5.82^	1994 - 1997	0.84	1998 - 2001	-11.67^	2002 - 2019	-2.10^
Canada	1985 - 1998	-0.27	1999 - 2001	-7.53	2002 - 2006	4.38^		
Croatia	1985 - 1988	-17.92^	1989 - 2005	1.82^	2006 - 2012	-10.24^	2013 - 2018	16.40^
Czech Republic	1986 - 1998	-4.59^	1999 - 2020	1.54^				
Denmark	1994 - 2001	-2.36	2002 - 2004	12.11	2005 - 2019	-0.66		
Estonia	1985 - 1992	-1.9	1993 - 1995	14.51	1996 - 2007	-5.75^	2008 - 2017	-0.41

Finland	1987 - 2003	-2.14^	2004 - 2006	-24.95^	2007 - 2009	-1.5	2010 - 2019	-10.83^
France	1985 - 1993	0.62	1994 - 2015	-1.63^				
Germany	1990 - 1998	-2.23^	1999 - 2005	4.63^	2006 - 2020	-1.20^		
Greece	1985 - 1991	-5.29^	1992 - 2008	3.97^	2009 - 2019	0.24		
Hungary	1985 - 2007	-4.22^	2008 - 2020	1.12				
Iceland	1985 - 1995	-0.65	1996 - 1998	-23.44	1999 - 2020	-1.80^		
Ireland	1985 - 1992	-3.23^	1993 - 1999	2.7	2000 - 2016	-5.77^		
Israel	1985 - 1991	-2.73	1992 - 1994	-21.22^	1995 - 1999	15.01^	2000 - 2019	1.30^
Italy	1985 - 1991	-8.78^	1992 - 2008	-0.02	2009 - 2018	6.15^		
Japan	1985 - 1994	1.55^	1995 - 2001	-3.05^	2002 - 2015	-0.92^	2016 - 2019	-13.58^
Latvia	1985 - 2019	-1.51^						
Lithuania	1985 - 1991	-5.26	1992 - 1995	11.96	1996 - 1998	-10.97	1999 - 2020	2.36^
Luxembourg	1985 - 1995	-6.32^	1996 - 2003	12.30^	2004 - 2019	-3.19^		
Malta	1985 - 1987	34.98	1988 - 2018	-1.62^				
Moldova	1985 - 2019	-2.32^						
Netherlands	1985 - 1988	-9.7	1989 - 1999	6.81^	2000 - 2019	-2.90^		
New Zealand	1985 - 1996	-4.30^	1997 - 1999	-19.46	2000 - 2017	1.91^		
Poland	1985 - 1992	-5.92^	1993 - 2019	1.05^				
Portugal	1985 - 2006	0.51	2007 - 2009	10.48	2010 - 2019	-1.67		

Romania	1985 - 1991	-4.32^	1992 - 1996	0.73	1997 - 2009	-5.53^	2010 - 2019	2.36^
Slovakia	1992 - 1999	-11.85^	2000 - 2015	1.46				
Slovenia	1985 - 2004	-0.49	2005 - 2020	-4.82^				
Spain	1985 - 1997	-2.75^	1998 - 2000	8.48	2001 - 2018	-0.37		
Sweden	1987 - 2004	-3.37^	2005 - 2019	-0.86^				
Switzerland	1995 - 1999	3.7	2000 - 2020	-1.76^				
United Kingdom	1985 - 1991	-2.55	1992 - 1994	17.4	1995 - 2017	-2.42^		
United States	1985 - 1988	2.98^	1989 - 1992	-1.7	1993 - 2002	0.66^	2003 - 2008	-1.79^
