# Supplementary material for: Multinational trends in sepsis mortality between 1985 and 2019: a temporal analysis of the WHO Mortality Database
Source: BMJ Open. 2024 Sep 12;14(9):e074822. doi: 10.1136/bmjopen-2023-074822 (PMC11404178; doi:10.1136/bmjopen-2023-074822)
Supplement: Supplementary file 2 [file bmjopen-2023-074822.reviewer_comments.pdf]

PEER REVIEW HISTORY

BMJ Open publishes all reviews undertaken for accepted manuscripts. Reviewers are asked to complete a checklist review form and are provided with free text boxes to elaborate on their assessment. These free text comments are reproduced below.

ARTICLE DETAILS

**TITLE (PROVISIONAL)**	Multinational trends in sepsis mortality between 1985 and 2019: a temporal analysis of the WHO mortality database.
**AUTHORS**	Komorowski, Matthieu; Salciccioli, Justin D; Shalhoub, Joseph; Gordon, Anthony; Marshall, Dominic C

VERSION 1 - REVIEW

**REVIEWER NAME**	Chua, Mui Teng
**REVIEWER AFFILIATION**	National University Hospital
**REVIEWER CONFLICT OF INTEREST**	No conflict of interest
**DATE REVIEW RETURNED**	01-Aug-2023

**GENERAL COMMENTS**	Thank you for the opportunity to review this excellent paper. I would suggest a statistician to review the statistics given the advanced regression analyses used.<br>For the limitations, it would be good to discuss the limitation in applicability even in high income countries given that these are aggregate data.

**REVIEWER NAME**	Artigas, Antonio
**REVIEWER AFFILIATION**	Sabadell Hospital, Spain, Critical Care Center
**REVIEWER CONFLICT OF INTEREST**	No competing interests
**DATE REVIEW RETURNED**	14-Aug-2023

**GENERAL COMMENTS**	This is a retrospective observational study of sepsis-related mortality using data deposited in the WHO Mortality Database over 3 decades in 36 countries.The authors reported a decrease in related mortality for both genders with a significant variability btween health systems and genders.Comments:<br>1.Countries: Data cannot be extrapolated to LMICs where sepsis and mortality is higher.The study covert 15,7% of world population. Is it a representative sample ?<br>2. Start data: What is the impact of start ASDR among countries ?Improvement could be higher in those countries with an initial higher ASDR.<br>3.Excessive missings: Please indicate what you consider as an excessive missing data for exclussion. >50% ?<br>4. Outcomes : was a mortality at day 60 or 90 day or..?<br>5.Cause of sepsis:can you include the cause of infection in septic patiens?

VERSION 1 – AUTHOR RESPONSE

Reviewer #1

Thank you for the opportunity to review this excellent paper.

Thank you for your encouraging words and for your insightful comments.

Comment #1:

I would suggest a statistician to review the statistics given the advanced regression analyses used.

Response #1:

The method we apply here has been used in a large number of publications by our group over the last 8 years, including, but not limited to: https://pubmed.ncbi.nlm.nih.gov/27006480/ ; https://pubmed.ncbi.nlm.nih.gov/29348182/ ; https://pubmed.ncbi.nlm.nih.gov/32133614/ ; https://pubmed.ncbi.nlm.nih.gov/30487157/ ; https://pubmed.ncbi.nlm.nih.gov/29728122/ ; https://pubmed.ncbi.nlm.nih.gov/32709465/ ; https://pubmed.ncbi.nlm.nih.gov/34551087/ ; https://pubmed.ncbi.nlm.nih.gov/26766741/ ; https://pubmed.ncbi.nlm.nih.gov/32391589/ ; https://pubmed.ncbi.nlm.nih.gov/34321515/ ; https://pubmed.ncbi.nlm.nih.gov/33640365/ ; https://pubmed.ncbi.nlm.nih.gov/34988220/.

As such, the method has been validated by a large number of statistical reviewers and we are confident in its robustness. We discuss at length the limitations of the approach, which pertain to challenges in data availability and quality.

The joinpoint regression method used is detailed in an index publication (reference #17: Kim HJ, Fay MP, Feuer EJ, Midthune DN. Permutation tests for joinpoint regression with applications to cancer rates. Stat Med (2000) 19:335–351). The software required to perform joinpoint regression is openly available (SEER*Stat software Version 8.3.6.0 provided by the United States National Cancer Institute Surveillance Research Program) and the analysis requires no tuning or selection of hyperparameters, meaning that the results are directly replicable by other researchers.

Comment #2:

For the limitations, it would be good to discuss the limitation in applicability even in high income countries given that these are aggregate data.

Response #2:

We appreciate the reviewer's insightful comment regarding the limitations in the applicability of our findings, even in high-income countries, due to the use of aggregate data. It is important to acknowledge that aggregate data have inherent limitations in capturing the nuances and complexities of individual patient experiences and healthcare delivery systems.

We have modified the following paragraph in the discussion to better capture this important point, L266-271 “We only presented aggregate national-level statistics. Sub-national trends may vary widely. While our results provide valuable insights at a population level and allow for the examination of broad trends over time, they do not capture the full spectrum of variation that exists within

populations, including differences in healthcare access, quality of care, socioeconomic factors, and regional disparities. As such, our findings should be interpreted with caution, particularly when considering their direct applicability to individual clinical practice or policy-making."

Moreover, while high-income countries generally have more robust data collection systems compared to low- and middle-income countries, there are still limitations in the accuracy and completeness of mortality data, as well as variations in coding practices and healthcare infrastructure across different regions within these countries. These factors may introduce biases and uncertainties into our analyses and may limit the generalizability of our findings to specific subpopulations or geographical areas. We acknowledge this in the discussion, L252-260.

Reviewer #2

This is a retrospective observational study of sepsis-related mortality using data deposited in the WHO Mortality Database over 3 decades in 36 countries. The authors reported a decrease in related mortality for both genders with a significant variability btween health systems and genders.

Thank you for your thorough and constructive feedback on our manuscript.

Comments:

Comment #1.

Countries: Data cannot be extrapolated to LMICs where sepsis and mortality is higher.The study covert 15,7% of world population. Is it a representative sample ?

Response #1

Thank you for this comment. This is a very important limitation which we acknowledge in the first paragraph of our results L166: "Mid-way through our analysis (in the year 2000), our data coverage represented about 15.7% of the world's population (971 million out of 6.2 billion individuals)."

Additionally, we specifically used the term "multinational" as opposed to "global" in the title of the article, to stress that the results could not be used to summarise world-wide trends.

In the discussion, we highlight how these results are difficult to extrapolate to LMICs, where over 80% of cases of sepsis occurs. The following statement can be found L272-275: "Finally, this approach does not capture low- and middle-income countries' (LMICs) data well, since many of these countries have major gaps in data recording. This limits the coverage of the study, since 87% of the world's population lives in LMICs. However, the validity of extrapolating data to these regions has been questioned (3,8,29)."

Still, our work represents one of the largest studies to date, which indirectly is an argument for improved mechanisms for monitoring sepsis incidence and outcomes, in line with the 2017 WHO Resolution "Recognizing Sepsis as a Global Health Priority", published in the NEJM, which we refer to in the introduction, reference #8.

Comment #2

Start data: What is the impact of start ASDR among countries ?Improvement could be higher in those countries with an initial higher ASDR.

Response #2

Thank for you this important suggestion. Below, we plot the relationship between the ASDR at the early period (averaged for the period 1985-1987) and the total relative change we measured (the percentage of change between the early period and the late period), separating male and female. We excluded 6 countries which did not have data available for the start and/or end period. As you hypothesized, we did observe a weak inverse correlation (R coefficient -0.50 ; R-squared 0.25) between the ASDR during the early study period (1985-1987) and the observed relative change over the whole study period. Countries with a higher early ASDR tend to be associated with a higher reduction in ASDR.

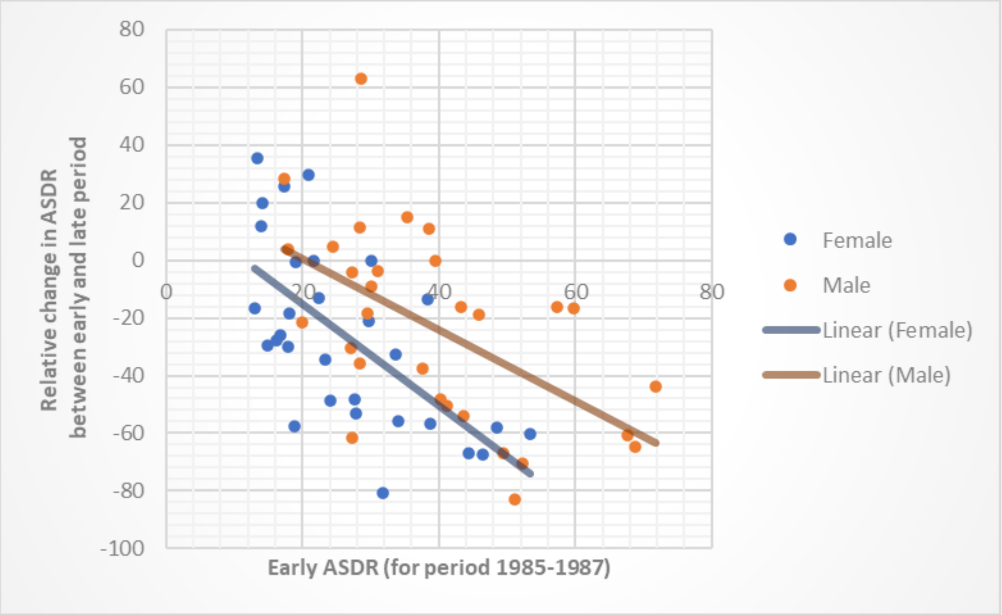

We included this figure in supplementary appendix (Supplementary Figure 2) and added the following paragraph to our result section (L176-179): “We measured a weak inverse correlation (R-squared 0.25) between the ASDR during the early study period (1985-1987) and the observed relative change over the whole study period: countries with a higher early ASDR tend to be associated with a higher reduction in ASDR (Supplementary Figure 2).”

Comment #3

Excessive missings: Please indicate what you consider as an excessive missing data for exclussion. >50% ?

Response #3

As stated in the methods (L 115-116), we excluded countries which did not have at least 10 years of available data during the period of interest (1985-2019), so we excluded countries with over 75% missingness (29 years divided by 39 years). The Online Resource 1 presents the list of the 50 countries with high usability data, specifying those which were included (N=36) or excluded (N=14) from the analysis based on this data missingness criterion.

Comment #4

Outcomes : was a mortality at day 60 or 90 day or..?

Response #4

Defining the mortality at a given time point (e.g. 60 or 90 days) requires the knowledge of an encounter with a healthcare institution. The mortality data used in the WHO registry is sourced from national civil registries, and is not attached to a particular hospital admission or clinical encounter. The following information is provided on the WHO website (https://www.who.int/news-room/questions-and-answers/item/who-mortality-database): "The cause-of-death statistics are from country civil registration systems. When a death occurs, this event is registered at the local civil registry with information on the cause of death. The information is then compiled by the national authority and submitted to WHO every year."

As such, the mortality we used in the analysis cannot be strictly defined as a mortality at a given time point. We hope that this explanation is understandable.

We added the following statement in the methods: L108-109 "Of note, deaths are not attached to a particular clinical encounter or hospital admission, so mortality cannot be defined at a given time point (e.g. "mortality at 90 days")."

Comment #5

Cause of sepsis: can you include the cause of infection in septic patiens?

Response #5:

Thank you for this valuable suggestion. In line with what you are suggesting, the sepsis abstraction we used for our main results (the Angus definition, coming from reference…) includes the ICD codes related to sepsis and septicaemia and also the root causes of sepsis (for example pneumonia, peritonitis, etc.).

[L243-246] "we have not restricted ICD codes to "sepsis" or "septicaemia" only but have also included the root causes of sepsis (such as pneumonia, peritonitis, etc.) following previously described definitions based on ICD coding and presented sensitivity analyses from several definitions."

[L253-256]: "In the Angus ICD abstraction of the sepsis definition, two criteria were required: a code for infection and a code for organ failure (21,30) Only the code for primary cause of death was available to us (as a suspected cause of death). We contend that since these patients died as a consequence of an infection, it is reasonable to assume that they indeed had sepsis and organ failure."

Again, we hope that these responses are satisfactory, and we thank the reviewers and editorial team for their time and effort.

VERSION 2 – REVIEW

**REVIEWER NAME**	Artigas, Antonio
**REVIEWER AFFILIATION**	Sabadell Hospital, Spain, Critical Care Center
**REVIEWER CONFLICT OF INTEREST**	I have no competing interest related to this study
**DATE REVIEW RETURNED**	12-Apr-2024

**GENERAL COMMENTS**	Thank you to answer precisely the comments of reviewers . I don't have additional comments

VERSION 2 – AUTHOR RESPONSE
